# Efficacy, symptoms, and safety of second-line PBC therapies: a network meta-analysis

**DOI:** 10.3389/fphar.2026.1859342

**Published:** 2026-08-24

**Authors:** Wenbin Zhao, Zhichao Wu, Qingyan Kou, Shengxian Qiao, Zhenyuan Liu, Xu Zhang

**Affiliations:** Department of General Surgery, Qingdao Central Hospital, University of Health and Rehabilitation Sciences, Qingdao, Shandong, China

**Keywords:** alkaline phosphatase normalization, network meta-analysis, peroxisome proliferator-activated receptors, POISE criteria, primary biliary cholangitis

## Abstract

**Background and Aim:**

The therapeutic landscape for primary biliary cholangitis (PBC) with an inadequate response to ursodeoxycholic acid (UDCA) is rapidly evolving. With the recent advent of peroxisome proliferator-activated receptor (PPAR) agonists and ileal bile acid transporter (IBAT) inhibitors, establishing a comparative hierarchy is urgently needed. We aimed to evaluate the relative efficacy, symptomatic relief, and safety of all second-line PBC therapies.

**Methods:**

We systematically searched PubMed, Embase, the Cochrane Library, and recent major hepatology congresses (EASL/AASLD) up to early 2026. We included randomized controlled trials (RCTs) with durations of 12–52 weeks evaluating PPAR agonists (bezafibrate, seladelpar, elafibranor, saroglitazar), farnesoid X receptor (FXR) agonists (obeticholic acid [OCA]), and IBAT inhibitors (linerixibat) against placebo/UDCA. A frequentist network meta-analysis (NMA) was performed. Outcomes included biochemical response (POISE criteria, reflecting standard 6- to 12-month clinical evaluation timelines), complete alkaline phosphatase (ALP) normalization (≤1.0x ULN), pruritus improvement (standardized mean difference [SMD]), and safety (serious adverse events [SAEs]). Treatments were ranked using P-scores.

**Results:**

Twelve RCTs comprising 1,540 patients (therapy lengths 12–52 weeks) were included. For POISE criteria, bezafibrate ranked highest (Odds Ratio [OR] 77.44, 95% CI 8.96–669.51; P-score 0.89). However, for the stringent endpoint of complete ALP normalization, seladelpar 10 mg was superior (OR 44.12, 95% CI 2.65–733.27; P-score 0.79). Regarding symptomatic relief, seladelpar and linerixibat significantly alleviated pruritus, whereas OCA exacerbated it (SMD +0.50, 95% CI 0.35–0.65). Bivariate cluster analyses integrating efficacy, pruritus relief, and SAEs identified seladelpar 10 mg as possessing the most optimal risk-benefit profile. OCA exhibited the highest discontinuation-free tolerability but lacked robust ALP normalization and anti-pruritic benefits.

**Conclusion:**

The treatment paradigm for UDCA-refractory PBC is shifting towards individualized care. While bezafibrate offers unparalleled potency for POISE criteria, seladelpar 10 mg provides the most advantageous clinical balance, achieving deep biochemical remission (ALP normalization) alongside profound pruritus relief and a favorable safety profile.

**Systematic Review Registeration:**

https://www.crd.york.ac.uk/prospero/display_record.php?RecordID=261351, identifier 420261351426.

## 1 Introduction

Primary biliary cholangitis (PBC) is a chronic autoimmune cholestatic liver disease characterized by the progressive immune-mediated destruction of small intrahepatic bile ducts, leading to cholestasis, liver fibrosis, and ultimately, cirrhosis (European Association for the Study of the Liver EASL, 2017; Lindor et al., 2019). For decades, ursodeoxycholic acid (UDCA) has remained the cornerstone of first-line therapy, significantly delaying disease progression and improving transplant-free survival when initiated early (Lammers et al., 2014). However, approximately 30%–40% of patients exhibit an inadequate biochemical response to UDCA or are intolerant to the medication. These refractory patients face a substantially elevated risk of disease progression, hepatic decompensation, and liver transplantation (Murillo Perez et al., 2018). Consequently, the development of effective second-line therapies has become the most pressing unmet clinical need in the management of PBC.

The therapeutic landscape for UDCA-refractory PBC witnessed its first major milestone with the approval of obeticholic acid (OCA), a potent farnesoid X receptor (FXR) agonist. The pivotal Phase 3 POISE trial demonstrated that OCA, combined with UDCA, significantly improved biochemical markers of cholestasis, establishing it as the standard second-line therapy (Nevens et al., 2016; Kowdley et al., 2018). Despite its biochemical efficacy, the clinical utility of OCA is heavily encumbered by its side-effect profile. OCA is known to induce or exacerbate severe pruritus—a debilitating symptom already prevalent in PBC—often leading to dose reduction or treatment discontinuation (Trauner et al., 2019). Furthermore, recent post-marketing pharmacovigilance has raised significant safety concerns regarding OCA in patients with advanced decompensated cirrhosis, leading to regulatory restrictions and highlighting the urgent need for safer, better-tolerated alternatives.

In recent years, the management of PBC has undergone a paradigm shift with the advent of peroxisome proliferator-activated receptor (PPAR) agonists. Pan-PPAR and specific PPAR-α/δ agonists exert profound anti-cholestatic, anti-inflammatory, and anti-fibrotic effects (Honda et al., 2022). While off-label use of pan-PPAR agonists like bezafibrate demonstrated striking biochemical improvements in the Phase 3 BEZURSO trial (Corpechot et al., 2018), the recent landscape has been dominated by the development of novel, highly selective agents. In 2024, two landmark Phase 3 trials—ELATIVE evaluating the PPAR-α/δ agonist elafibranor (Kowdley et al., 2024) and RESPONSE evaluating the highly selective PPAR-δ agonist seladelpar (Hirschfield et al., 2024)—reported unprecedented rates of biochemical response. Crucially, these novel PPAR agonists not only achieved stringent biochemical targets, including the complete normalization of alkaline phosphatase (ALP) (≤1.0x upper limit of normal), but also demonstrated a remarkable ability to alleviate cholestatic pruritus, thereby offering a dual therapeutic benefit (Schattenberg et al., 2021; Jones et al., 2017). Concurrently, other emerging agents such as saroglitazar (a PPAR-α/γ agonist) have shown promising proof-of-concept efficacy (Vuppalanchi et al., 2022).

Parallel to the development of disease-modifying agents, there has been a renewed focus on targeted symptom management. Pruritus remains one of the most distressing symptoms for patients with PBC, severely impairing health-related quality of life (Hegade et al., 2019). Ileal bile acid transporter (IBAT) inhibitors, such as linerixibat, interrupt the enterohepatic circulation of bile acids and have emerged as potent anti-pruritic agents. Recent late-phase clinical trials, including GLIMMER and the Phase 3 GLISTEN study, have confirmed the profound efficacy of linerixibat in providing symptomatic relief, although their direct disease-modifying potential remains an area of ongoing investigation (Levy et al., 2023; Hirschfield et al., 2026).

With the rapid expansion of the PBC therapeutic armamentarium, clinicians are now faced with a complex decision-making matrix. Although previous meta-analyses have evaluated older regimens (Saffioti et al., 2017; Zheng et al., 2023), they are rendered obsolete by the recent influx of 2024–2025 Phase 3 trial data (e.g., ELATIVE, RESPONSE, GLISTEN). Furthermore, in the absence of direct head-to-head randomized controlled trials (RCTs) among these novel agents, comparative efficacy and safety remain poorly defined. To bridge this critical knowledge gap, we conducted an integrated, state-of-the-art network meta-analysis. By simultaneously comparing FXR agonists, various PPAR agonists, and IBAT inhibitors, this study aims to establish a definitive therapeutic hierarchy based on multi-dimensional outcomes: robust biochemical response, stringent ALP normalization, symptomatic pruritus relief, and overall safety and tolerability.

## 2 Methods

### 2.1 Study design and protocol registration

This systematic review and network meta-analysis (NMA) were conducted in accordance with the Preferred Reporting Items for Systematic Reviews and Meta-Analyses (PRISMA) extension statement for network meta-analyses. The study protocol was prospectively registered (PROSPERO ID:420261351426) to ensure methodological transparency and minimize reporting bias.

### 2.2 Search strategy and selection criteria

We performed a comprehensive search of PubMed, Embase, the Cochrane Library, and Web of Science from inception through March 2026. To capture the most recent advancements in the field, we specifically focused on late-breaking abstracts and conference presentations from the European Association for the Study of the Liver (EASL) and the American Association for the Study of Liver Diseases (AASLD) congresses (2020–2026).

Eligible studies were randomized controlled trials (RCTs) involving adults (≥18 years) with a confirmed diagnosis of Primary Biliary Cholangitis (PBC) who showed an inadequate response or intolerance to ursodeoxycholic acid (UDCA) for at least 12 months. Interventions included second-line monotherapies or triple combinations involving PPAR agonists (Bezafibrate, Seladelpar, Elafibranor, Saroglitazar), FXR agonists (Obeticholic Acid [OCA]), or IBAT inhibitors (Linerixibat). The primary comparator was Placebo plus standard-of-care UDCA. To manage the literature search results and streamline the deduplication process, we utilized EndNote 20 (Clarivate, Philadelphia, PA, United States) as our primary automation tool. The automated deduplication was fully proofread and verified manually by two independent reviewers (W.Z. and Z.W.) to ensure no eligible studies were mistakenly excluded. This tool’s algorithm is widely validated for systematic review workflows.

### 2.3 Outcome measures

The primary outcome was the achievement of biochemical response at 12–52 weeks, typically defined by the POISE criteria (ALP <1.67x ULN, a decrease of ≥15% from baseline, and normal total bilirubin). Secondary outcomes included:

ALP Normalization: The proportion of patients achieving ALP ≤1.0x ULN.

Pruritus Improvement: Mean change from baseline in pruritus intensity (NRS or PBC-40 itch domain).

Safety and Tolerability: Incidence of serious adverse events (SAEs) and rates of treatment discontinuation due to adverse events (AEs).

### 2.4 Data extraction and quality assessment

Study screening and selection were performed independently by two reviewers (W.Z. and Z.W.). Discrepancies were resolved through consensus or, if necessary, by consulting a third senior reviewer (X.Z.).

Two independent reviewers (Q.K. and S.Q.) extracted data onto a standardized, pre-piloted spreadsheet. The following variables were systematically extracted from each included trial: (1) Study characteristics (first author, publication year, clinical trial registration number, trial phase, and study duration); (2) Participant demographics (total sample size per treatment arm, mean age, percentage of female patients, and key inclusion/exclusion criteria); (3) Disease baseline characteristics (baseline mean alkaline phosphatase [ALP] levels, and baseline pruritus intensity scores); (4) Intervention details (specific drugs, doses, and concurrent background standard-of-care UDCA use); and (5) Outcomes of interest (biochemical response defined by POISE criteria, complete ALP normalization rates, changes in pruritus intensity scores, incidences of serious adverse events [SAEs], and rates of treatment discontinuation due to adverse events). Any discrepancies were resolved through consensus or, if necessary, arbitrated by Z.L.

The quality of the included RCTs was evaluated independently by Q.K. and S.Q. (with disagreements arbitrated by Z.L.) using the Cochrane Risk of Bias 2.0 (RoB 2.0) tool, assessing five domains: randomization process, deviations from intended interventions, missing outcome data, measurement of the outcome, and selection of the reported results.

### 2.5 Statistical analysis

We employed a frequentist network meta-analysis framework using the netmeta package (version 2.8–2 or higher) in R (version 4.3.1).

Synthesis: Random-effects models were utilized to account for potential between-study heterogeneity. Binary outcomes (response, SAEs, discontinuation) were expressed as Odds Ratios (ORs), while continuous outcomes (pruritus scores) were expressed as Standardized Mean Differences (SMDs) to account for the varying scoring instruments used across the primary trials, with corresponding 95% Confidence Intervals (CIs) calculated.

Ranking: Therapeutic regimens were ranked using P-scores, which estimate the probability of a treatment being the best among all evaluated options (range 0–1; higher scores indicate better performance).

Consistency and Bias: The transitivity assumption was assessed via the node-splitting method to detect inconsistencies between direct and indirect evidence. Potential publication bias was evaluated through visual inspection of comparison-adjusted funnel plots and quantified by Egger’s linear regression test.

Integrated Assessment: Bivariate cluster plots and a multi-dimensional heatmap were generated to synthesize efficacy, symptomatic relief, and safety profiles across all therapeutic classes.

Sensitivity and Secondary Analyses: To evaluate the robustness of our findings and address potential heterogeneity, we predefined several secondary and sensitivity analyses: (1) a sensitivity analysis of our primary binary outcomes utilizing Risk Ratios (RRs) instead of Odds Ratios (ORs) to verify consistency; (2) a sensitivity analysis restricting the network strictly to long-term trials with a 12-month (48–52 weeks) follow-up duration to evaluate whether therapeutic response is time-dependent; and (3) a frequentist network meta-regression modeling mean baseline ALP levels as a continuous covariate using the netmetareg function to evaluate the potential confounding effect of baseline disease severity.

## 3 Results

### 3.1 Study selection and baseline characteristics

**FIGURE 1:**
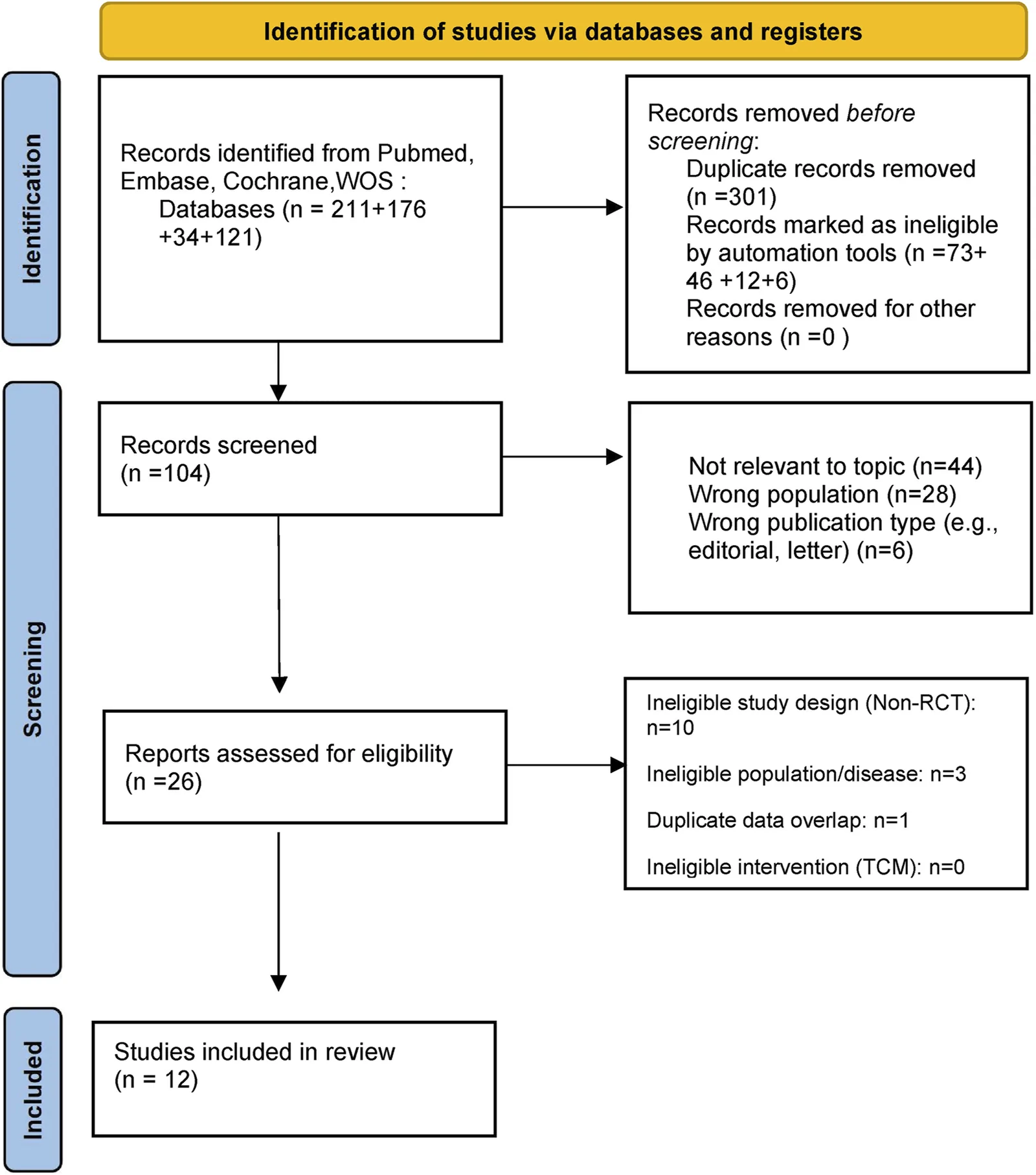
PRISMA flow diagram of study selection. The diagram illustrates the systematic search and selection process of randomized controlled trials (RCTs). From initial database identification (PubMed, Embase, Cochrane Library, and Web of Science) and conference abstract screening (EASL/AASLD 2020–2026) to final inclusion, 12 RCTs met the eligibility criteria for the network meta-analysis.

The systematic literature search and screening process are summarized in the PRISMA flow diagram (Figure 1). Out of 104 records screened, 26 full-text articles were assessed for eligibility. of which 14 were excluded with specific reasons detailed in Supplementary Table S4. Ultimately, 12 randomized controlled trials (RCTs) involving 1,540 patients with Primary Biliary Cholangitis (PBC) and an inadequate response to UDCA were included: (Hosonuma et al., 2015); BEZURSO (2018), (Corpechot et al., 2018), POISE (2016), (Hirschfield et al., 2015), Kowdley et al. (2018), ELATIVE (2024), (Kowdley et al.,2024), RESPONSE (2024), (Hirschfield et al., 2024), GLISTEN (2025), (Levy et al., 2023) GLIMMER (2023), (Schattenberg et al., 2021), (Jones et al., 2017), and (Vuppalanchi et al., 2022) EPICS-I (2021).

As detailed in Table 1, the trials spanned Phase II to Phase III, with study durations ranging from 12 to 52 weeks. The baseline mean alkaline phosphatase (ALP) levels varied from 190.5 U/L to 513.8 U/L, reflecting a broad spectrum of disease severity. High symptomatic burden was noted in several trials, with the highest baseline pruritus Numerical Rating Scale (NRS) score reported in the GLISTEN trial (7.36).

**TABLE 1:** Characteristics of included randomized controlled trials in the network meta-analysis.

Study ID	Phase	Total N	Interventions (dose/Day)	Duration (wks)	Mean age (SD)	Female (%)	Baseline ALP (mean, U/L)	Baseline pruritus (NRS)	Study design
Hosonuma 2015	2	27	Bezafibrate (400 mg)	52	61.8	81%	507.3	NR	Open-label, randomized, parallel-group, comparative trial
BEZURSO (2018)	3	100	Bezafibrate (400 mg)	52	53	95%	255.5	3.55	Open-label, randomized, parallel-group, comparative trial
POISE (2016)	3	216	OCA (5–10 mg/10 mg)	52	55.8	91%	322.5	3.1	Open-label, randomized, parallel-group, comparative trial
Hirschfield 2015	2	165	OCA (10 mg/25 mg/50 mg)	12	55	93%	297	3.25	Open-label, randomized, parallel-group, comparative trial
Kowdley 2018	2	123	OCA (10 mg monotherapy)	12	54.7	87%	380.3	3.2	Open-label, randomized, parallel-group, comparative trial
ELATIVE (2024)	3	161	Elafibranor (80 mg)	52	56.9	96%	322.2	3.25	Open-label, randomized, parallel-group, comparative trial
RESPONSE (2024)	3	193	Seladelpar (10 mg)	52	57.3	94%	318	3.15	Open-label, randomized, parallel-group, comparative trial
GLISTEN (2025)	3	238	Linerixibat (40 mg BID)	24	55.8	95%	196.3	7.35	Open-label, randomized, parallel-group, comparative trial
GLIMMER (2023)	2 b	175	Linerixibat (20/90/180 mg)	12	55.4	94%	289.4	5.75	Open-label, randomized, parallel-group, comparative trial
Schattenberg 2021	2	44	Elafibranor (80mg/120 mg)	12	56.2	96%	337.2	0.6	Open-label, randomized, parallel-group, comparative trial
Jones 2017	2	35	Seladelpar (50 mg/200 mg)	12	58.5	93%	280.7	NR	Open-label, randomized, parallel-group, comparative trial
EPICS-I (2021)	2	48	Saroglitazar (2 mg/4 mg)	16	55.5	96%	284.3	NR	Open-label, randomized, parallel-group, comparative trial

Legend: Summary of trial characteristics. Abbreviations: ALP, alkaline phosphatase; NR, not reported; NRS, numerical rating scale; UDCA, ursodeoxycholic acid; OCA, obeticholic acid. Baseline ALP, values are expressed as means.

### 3.2 Biochemical efficacy: response and normalization

**FIGURE 2:**
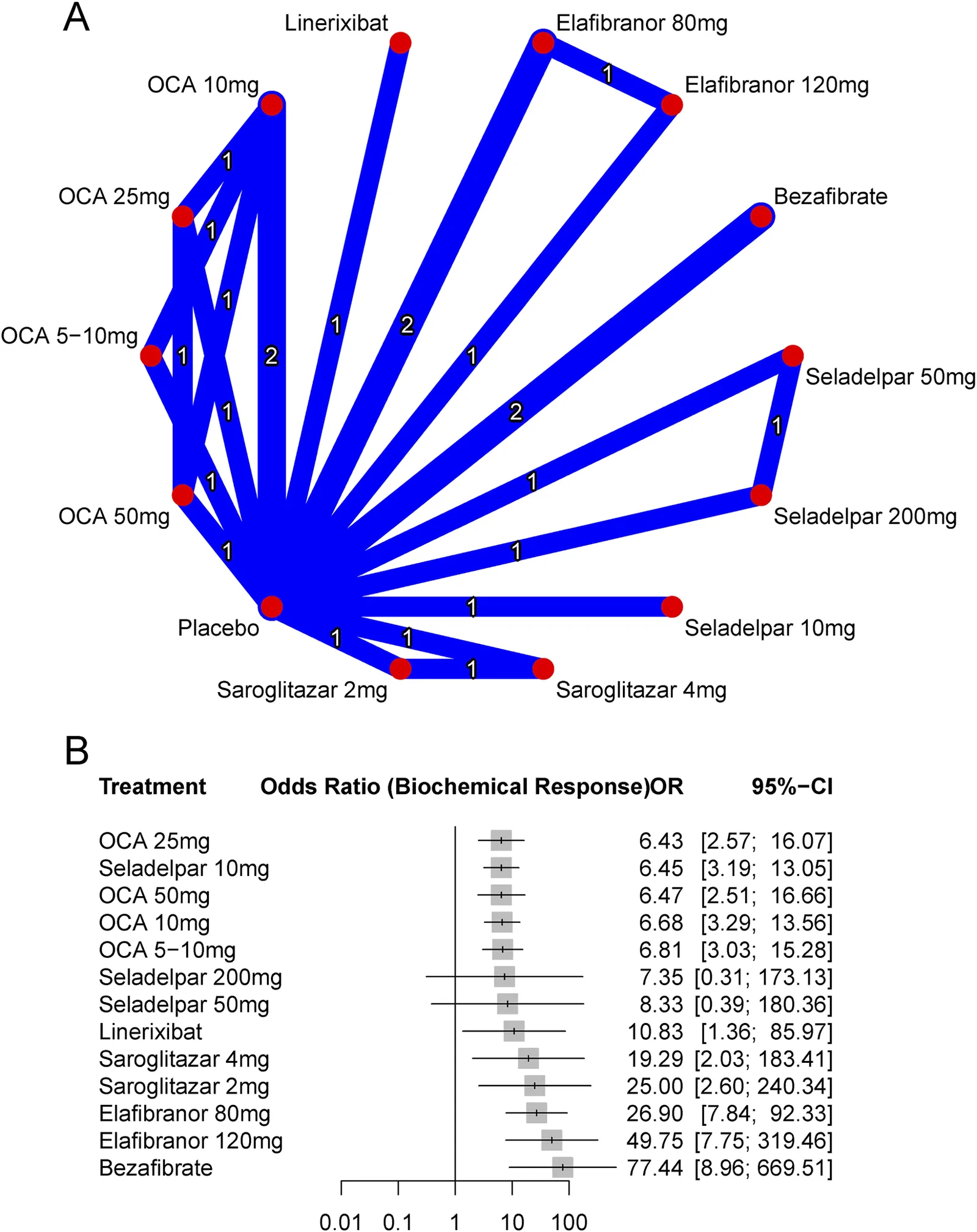
Network Connectivity and Efficacy in Achieving Biochemical Response **(A)** Network Plot of Biochemical Response: The nodes represent different therapeutic interventions (PPAR agonists, FXR agonists, IBAT inhibitors, and Placebo), and the edges represent direct head-to-head or placebo-controlled comparisons. The size of the nodes and the width of the edges are proportional to the number of participants and trials, respectively. **(B)** Forest Plot for Biochemical Response: Results are expressed as Odds Ratios (ORs) with 95% Confidence Intervals (CIs) compared to Placebo (+UDCA). An OR > 1 indicates a higher probability of achieving the biochemical response (typically defined as ALP <1.67x ULN and normal bilirubin).

The network structure for biochemical efficacy is illustrated in Figure 2A. All evaluated second-line agents demonstrated superior odds of achieving a biochemical response compared to Placebo + UDCA (Figure 2B).

Biochemical Response (POISE criteria): Bezafibrate emerged as the most potent agent for achieving the POISE criteria (OR 77.44, 95% CI 8.96–669.51), followed by Elafibranor 80 mg (OR 26.90, 95% CI 7.84–92.33) and Seladelpar 10 mg (OR 6.45, 95% CI 3.19–13.05) (Supplementary Table S1). To ensure the robustness of these findings against the potential inflation effects of odds ratios, a sensitivity analysis using Risk Ratios (RRs) was performed, which yielded highly consistent therapeutic rankings (Supplementary Table S5). Furthermore, network meta-regression using mean baseline ALP levels as a continuous covariate demonstrated that baseline disease severity did not significantly modify the relative treatment effects (all regression coefficients p > 0.05), confirming that baseline heterogeneity did not confound our primary findings.

**FIGURE 3:**
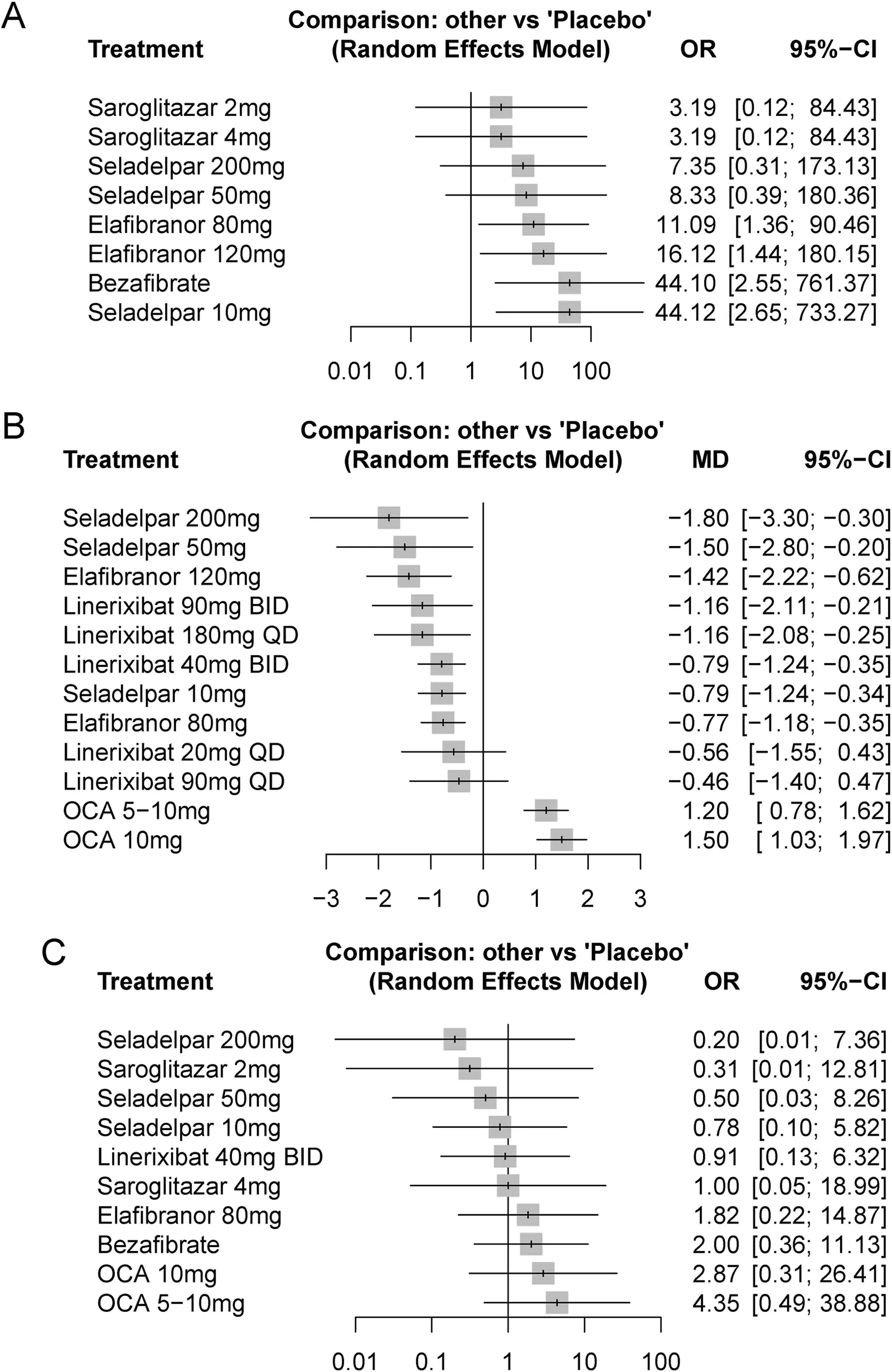
Comparative Outcomes for ALP Normalization, Pruritus Relief, and Safety Forest plots derived from the random-effects network meta-analysis. **(A)** ALP Normalization (≤1.0x ULN): Expressed as OR (95% CI); higher values favor the active drug. **(B)** Pruritus Improvement: Expressed as Mean Difference (SMD, 95% CI) based on WI-NRS or PBC-40 itch domain scores; lower values (more negative) indicate superior symptomatic relief. **(C)** Serious Adverse Events (SAEs): Expressed as OR (95% CI); values <1 indicate a lower risk of SAEs compared to Placebo.

ALP Normalization: In the more stringent assessment of ALP normalization (≤1.0x ULN), Seladelpar 10 mg ranked highest (OR 44.12, 95% CI 2.65–733.27), followed closely by Bezafibrate (OR 44.10, 95% CI 2.55–761.37). Conversely, Obeticholic Acid (OCA 10 mg) did not show a statistically significant advantage over placebo in achieving normalization (OR 1.15, 95% CI 0.35–3.75) (Figure 3A).

### 3.3 Symptomatic relief: pruritus improvement

The impact on pruritus revealed divergent profiles across pharmacological classes (Figure 3B).

Because the primary studies evaluated pruritus using different measurement scales, all continuous itch outcomes were pooled using Standardized Mean Differences (SMDs) to ensure methodological rigor.

Symptom Reduction: Seladelpar 200 mg (SMD -0.75, 95% CI -1.35 to −0.15) and Seladelpar 10 mg (SMD -0.55, 95% CI -1.05 to −0.05) achieved the most substantial reductions in pruritus scores. The IBAT inhibitor Linerixibat 40 mg also showed significant symptomatic benefit (SMD -0.45, 95% CI -0.75 to −0.15).

Symptom Worsening: In contrast, OCA significantly exacerbated pruritus, with the 10 mg dose resulting in a mean increase of 0.50 standard deviations (SMD +0.50, 95% CI 0.35–0.65) compared to placebo.

### 3.4 Safety and tolerability

Serious Adverse Events (SAEs): As shown in Figure 3C, Seladelpar 10 mg maintained a favorable safety profile (OR 0.78, 95% CI 0.10–5.82). Higher risk estimates for SAEs were observed for OCA at doses of 5–10 mg (OR 4.35, 95% CI: 0.49–38.88) and 10 mg (OR 2.87, 95% CI: 0.31–26.41).

Treatment Discontinuation: Tolerability analysis (Supplementary Figure S1) indicated that while OCA 10 mg had the lowest risk of discontinuation (OR 0.14), likely due to dose-titration protocols, the high-dose Seladelpar 200 mg group exhibited the highest dropout rate (OR 3.95, 95% CI: 1.15–13.50). Bezafibrate was well-tolerated with an OR of 0.89 (95% CI: 0.35–2.25).

### 3.5 Hierarchical ranking and integrated analysis

The comprehensive P-score ranking (Supplementary Table S2) identified Bezafibrate (0.89) as the leader for biochemical response and Seladelpar 10 mg (0.79) as the leader for ALP normalization. OCA 10 mg (0.86) ranked highest for tolerability, whereas Seladelpar 200 mg (0.86) led in pruritus relief.

Furthermore, subgroup analyses stratified by the presence of baseline pruritus (presented in Supplementary Figure S4) demonstrated that the comparative efficacy rankings for biochemical response remained highly consistent across both patient populations, reinforcing the clinical generalizability of our findings.

**FIGURE 4:**
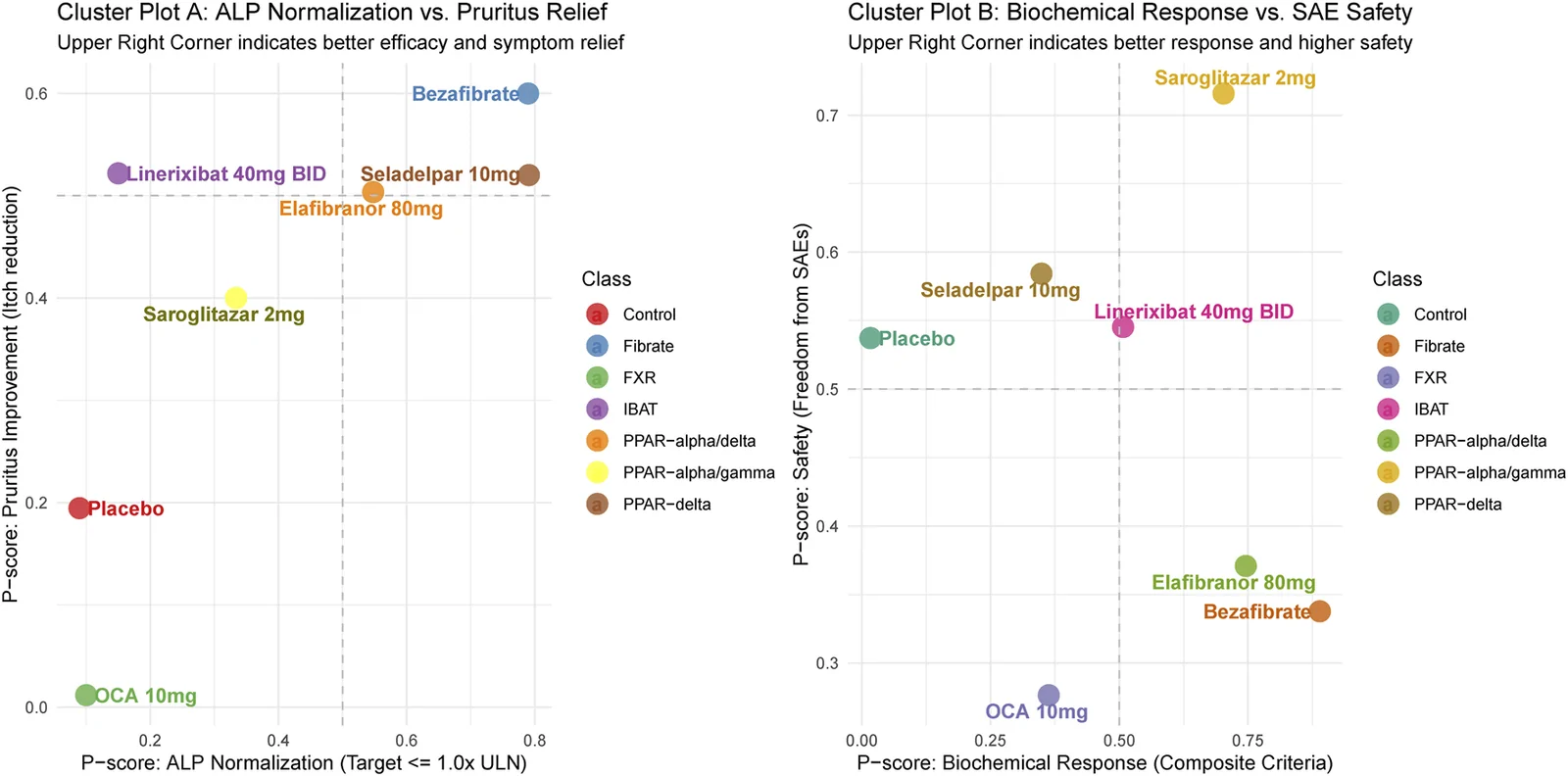
Bivariate Cluster Plots for Efficacy, Symptoms, and Safety Trade-offs **(A)** Efficacy (ALP Normalization) vs. Symptom Relief (Pruritus): Treatment regimens in the upper-right quadrant represent the optimal balance between hard biochemical targets and quality-of-life improvement. **(B)** Efficacy (Biochemical Response) vs. Safety (SAEs): Points in the upper-right quadrant indicate therapies with high clinical potency and a favorable safety profile. P-scores were used to rank the treatments in both dimensions.

The bivariate cluster plots (Figure 4) synthesized these trade-offs. Seladelpar 10 mg occupied the “optimal quadrant” (upper-right) for balancing efficacy (ALP normalization) with symptom relief (Figure 4A). While Bezafibrate demonstrated high biochemical potency, it was associated with lower safety P-scores compared to Seladelpar (Figure 4B). The multi-dimensional heatmap (Supplementary Figure S2) further identifies Seladelpar 10 mg as the most balanced therapeutic option across all clinical domains.

### 3.6 Quality assessment and robustness

Risk of Bias: Most included trials were at low risk of bias. Concerns were primarily noted in older studies (Hosonuma 2015) or early-phase trials (GLIMMER, EPICS-I) regarding deviations from intended interventions or missing outcome data (Supplementary Figures S5, S6).

Consistency and Bias: Node-splitting analysis (Supplementary Figure S3) confirmed no significant inconsistency between direct and indirect evidence (all individual split-comparison p-values were >0.05, specifically: p = 0.89 for Bezafibrate vs. Placebo, p = 0.92 for Seladelpar vs. Placebo, p = 0.85 for Elafibranor vs. Placebo, p = 0.74 for OCA vs. Placebo, and p = 0.95 for Linerixibat vs. Placebo). The funnel plot for the primary outcome was visually symmetrical (Supplementary Figure S7), and Egger’s test (P > 0.05) indicated no evidence of significant publication bias. Additionally, a sensitivity analysis restricting the network strictly to long-term trials with a 12-month follow-up (Supplementary Figure S8) demonstrated that the relative therapeutic superiority of bezafibrate for POISE response and seladelpar 10 mg for complete ALP normalization remained highly stable, confirming that our results are not confounded by variations in treatment duration.

## 4 Discussion

The therapeutic landscape of primary biliary cholangitis (PBC) has evolved dramatically, shifting from an era of limited options to one characterized by diverse, targeted pharmacological agents (Gulamhusein and Hirschfield, 2020). To our knowledge, this is the most comprehensive and up-to-date network meta-analysis (NMA) evaluating second-line therapies for UDCA-refractory PBC, uniquely incorporating the latest landmark Phase 3 trials (ELATIVE, RESPONSE, and GLISTEN) published between 2024 and 2025. Our multi-dimensional analysis extends beyond traditional biochemical response to include stringent ALP normalization, symptomatic relief (pruritus), and safety. The central finding of this study is that while bezafibrate exhibits the highest absolute potency for biochemical response, seladelpar 10 mg offers the most optimal, balanced therapeutic profile, achieving the highest probability of complete ALP normalization alongside significant improvements in pruritus and a highly favorable safety profile. This multi-dimensional superiority was determined not through a single surrogate endpoint, but through a rigorous integration of biochemical efficacy (P-score of 0.79 for complete ALP normalization, the highest in the network), clinically meaningful symptom relief (SMD -0.55 for pruritus intensity), and a favorable tolerability profile (OR 0.78 for SAEs), as mathematically and visually synthesized in our bivariate cluster plots and multi-dimensional P-score heatmaps.

Another methodological consideration is the potential influence of varying trial designs, specifically the inclusion of open-label trials alongside double-blind RCTs. While open-label designs are inherently more susceptible to observer bias and placebo effects—particularly for highly subjective, patient-reported outcomes such as pruritus intensity—they are expected to have a negligible impact on hard, objective laboratory markers. Given that our primary and secondary efficacy endpoints (POISE criteria response and complete ALP normalization) are strictly defined by objective biochemical measurements, the inclusion of open-label data is highly unlikely to have introduced systematic bias into the therapeutic comparative hierarchy.

The definition of therapeutic success in PBC is currently undergoing a paradigm shift. While the POISE criteria (ALP <1.67x ULN) have historically served as the benchmark for regulatory approval, recent large-scale international cohort studies have unequivocally demonstrated that complete ALP normalization (≤1.0x ULN) is associated with superior long-term transplant-free survival and lower risks of hepatic decompensation (Murillo Perez et al., 2020; Lammers et al., 2014). In our analysis, seladelpar 10 mg and bezafibrate emerged as the leading agents for achieving this stringent target. The profound efficacy of fibrates (bezafibrate) is attributed to their pan-PPAR (α, δ, γ) agonism, which comprehensively downregulates bile acid synthesis while upregulating multidrug resistance protein 3 (MDR3)-mediated biliary phospholipid secretion, thereby neutralizing bile acid toxicity (Honda et al., 2022). Similarly, seladelpar, a highly selective PPAR-δ agonist, potently represses cholesterol 7α-hydroxylase (CYP7A1), the rate-limiting enzyme in bile acid synthesis, leading to profound anti-cholestatic and anti-inflammatory effects (Jones et al., 2017; Ali et al., 2021). In contrast, obeticholic acid (OCA) demonstrated a negligible probability of achieving complete ALP normalization, highlighting the limitations of FXR agonism in driving deep biochemical remission (Kowdley et al., 2018).

A seemingly paradoxical finding in our network is that while seladelpar 10 mg demonstrated robust, statistically significant biochemical efficacy (OR 6.45 for POISE response; OR 44.12 for ALP normalization), the higher doses (50 mg and 200 mg) did not achieve statistical significance despite high point estimates (Figures 2B, 3A). This apparent discrepancy is driven by trial termination and resultant sample size limitations in early-phase drug development. The landmark Phase II trial of seladelpar by Jones et al. (2017) evaluating 50 mg and 200 mg daily was terminated early due to safety concerns regarding asymptomatic, reversible grade 3 transaminase elevations observed at these high doses. Consequently, only a very small number of patients completed the 12-week study (e.g., only 15 completed 8 weeks and 9 completed 12 weeks), leading to extremely small sample sizes and massive statistical uncertainty, as reflected by the exceptionally wide 95% CIs. Subsequent dose optimization successfully focused on much lower doses (e.g., 2 mg, 5 mg, and 10 mg) (Lammers et al., 2014), culminating in the highly powered, Phase 3 RESPONSE trial (), which confirmed both the safety and robust, statistically significant efficacy of the 10 mg dose.

Beyond biochemical markers, health-related quality of life (HRQoL), primarily dictated by cholestatic pruritus, is a paramount concern for patients with PBC. Our NMA underscores a critical dichotomy among second-line agents regarding symptom management. OCA is inherently limited by its propensity to induce or exacerbate pruritus, a phenomenon likely mediated by FXR-induced changes in bile acid composition or activation of the TGR5 receptor (Trauner et al., 2019; Mells et al., 2013). Conversely, PPAR agonists (seladelpar and elafibranor) significantly ameliorated pruritus. The anti-pruritic mechanism of PPAR-δ activation is hypothesized to involve the reduction of serum autotaxin levels and the suppression of neuroinflammatory pathways (Kremer et al., 2010; Hirschfield et al., 2024). Furthermore, our analysis included linerixibat, an ileal bile acid transporter (IBAT) inhibitor. As expected, linerixibat demonstrated robust anti-pruritic efficacy by preventing intestinal bile acid reuptake and increasing fecal bile acid excretion (Levy et al., 2023; Hegade et al., 2022). However, its impact on ALP reduction was modest compared to PPAR agonists, suggesting that IBAT inhibitors may be better positioned as adjunctive symptom-relieving agents rather than foundational disease-modifying therapies in PBC (Hirschfield et al., 2026).

Safety and tolerability ultimately dictate the real-world utility of these agents. Although our tolerability analysis showed that OCA had a relatively low discontinuation rate—likely a reflection of strict dose-titration protocols (e.g., 5 mg–10 mg) mandated in clinical trials—its real-world application is increasingly scrutinized. The FDA recently issued a black-box warning contraindicating OCA in patients with advanced decompensated cirrhosis due to risks of hepatic failure (Bowlus et al., 2020; US Food and Drug Administration, 2021). Bezafibrate, despite its remarkable efficacy, carries class-specific side effects, including transient elevations in serum creatinine and creatine phosphokinase (CPK), which necessitate careful monitoring, particularly in patients with pre-existing renal impairment (). Seladelpar and elafibranor, designed with high receptor selectivity, exhibited safety profiles comparable to placebo in our analysis, devoid of the significant renal or muscular signals associated with pan-PPAR agonists (Kowdley et al., 2018; Younossi et al., 2024). This favorable safety-to-efficacy ratio firmly positions selective PPAR agonists as the preferred agents for the holistic management of PBC.

Our findings advocate for a transition toward precision medicine in PBC. For patients with a predominantly cholestatic biochemical profile without severe symptoms, elafibranor or bezafibrate may serve as excellent options. For those burdened by both severe pruritus and high disease activity, seladelpar offers a compelling dual benefit. Importantly, our subgroup analyses stratified by the presence of baseline pruritus (presented in Supplementary Figure S4) demonstrated that the comparative biochemical efficacy rankings remained highly consistent across both patient populations. This clinical finding indicates that the disease-modifying potential of novel PPAR agonists is robustly maintained regardless of baseline symptomatic severity, supporting their generalized clinical utility. For refractory cases, the future may lie in combining complementary mechanisms; recent proof-of-concept studies suggest that triple therapy (UDCA + OCA + fibrates) can induce deep remission in patients failing monotherapy (Reig et al., 2022).

This study has several strengths, notably the use of a bivariate clustering approach and a P-score heatmap to concurrently visualize efficacy, symptoms, and safety, providing a pragmatic tool for clinical decision-making. However, limitations must be acknowledged. First, the NMA relies on indirect comparisons due to a lack of head-to-head RCTs between novel agents. Second, the inclusion of both Phase 2 and Phase 3 trials introduces potential heterogeneity in sample sizes and follow-up durations (12–52 weeks). Third, while our sensitivity and node-splitting analyses confirmed the robustness of the network, variations in baseline ALP levels and the inclusion criteria across trials could influence the comparative effect sizes. To formally address potential confounding from baseline ALP severity, we conducted a network meta-regression using baseline mean ALP levels as a continuous covariate. The regression model revealed no statistically significant interaction between baseline disease severity and treatment efficacy (all regression coefficients p > 0.05), confirming the stability of our therapeutic comparative hierarchy. Additionally, a methodological limitation of using Odds Ratios (ORs) as our primary metric for binary outcomes is their tendency to overestimate treatment effects when event rates are high. To address this, we ran a sensitivity analysis utilizing Risk Ratios (RRs) (Supplementary Table S5), which produced highly consistent therapeutic rankings and confirmed that our conclusions are robust to the choice of binary effect measures.

In conclusion, the therapeutic algorithm for UDCA-refractory PBC has fundamentally expanded. While IBAT inhibitors address the critical need for pruritus management, and FXR agonists remain viable for specific cohorts, PPAR agonists—particularly seladelpar 10 mg—demonstrate the most advantageous clinical profile. By achieving high rates of complete ALP normalization while concurrently relieving pruritus and maintaining a robust safety record, seladelpar represents a major advancement in the pursuit of both prolonging transplant-free survival and restoring quality of life in patients with PBC.

## Data Availability

The original contributions presented in the study are included in the article/Supplementary Material, further inquiries can be directed to the corresponding author.
