# Supplementary material for: Efficacy, symptoms, and safety of second-line PBC therapies: a network meta-analysis
Source: Front Pharmacol. 2026 Aug 24;17:1859342. doi: 10.3389/fphar.2026.1859342 (PMC13547053; doi:10.3389/fphar.2026.1859342)
Supplement: Supplementary file 1 [file Supplementaryfile1.docx]

**Efficacy, Symptoms, and Safety of Second-Line PBC Therapies: A Network Meta-Analysis**

**Supplementary**

| Intervention | Biochemical Response (OR, 95% CI) | ALP Normalization (OR, 95% CI) | Pruritus Improvement (MD, 95% CI) | Serious Adverse Events (OR, 95% CI) |
| --- | --- | --- | --- | --- |
| Bezafibrate | 12.45 (6.20–25.10)* | 8.12 (3.45–19.22)* | -1.10 (-1.85, -0.35)* | 1.15 (0.65, 2.05) |
| Seladelpar 10mg | 6.88 (4.12–11.50)* | 10.45 (5.12–21.30)* | -1.50 (-2.10, -0.90)* | 0.85 (0.42, 1.72) |
| Elafibranor 80mg | 8.15 (4.55–14.60)* | 5.20 (2.15–12.55)* | -0.95 (-1.65, -0.25)* | 1.45 (0.78, 2.70) |
| Saroglitazar 2mg | 7.20 (2.10–24.70)* | 2.55 (0.45–14.30) | -0.80 (-1.90, 0.30) | 0.65 (0.15, 2.85) |
| OCA 10mg | 3.42 (1.85–6.32)* | 1.15 (0.35–3.75) | +1.56 (0.85, 2.27)* | 1.95 (1.10, 3.45)* |
| Linerixibat 40mg | 2.15 (0.95–4.85) | 1.05 (0.22–4.95) | -1.25 (-1.95, -0.55)* | 0.95 (0.55, 1.65) |

Supplementary Table 1. Comparative Efficacy and Safety of Second-line Therapies vs. Placebo/UDCA

Supplementary Table 1 Legend: Results of the Network Meta-analysis comparing active agents against Placebo (+UDCA). OR > 1 favors active drug for efficacy; MD < 0 favors active drug for pruritus improvement. Asterisks () indicate statistical significance (95% CI does not cross the null value). Data derived from random-effects model.*

| Rank | Biochemical Response | ALP Normalization | Pruritus Relief | Safety (No SAEs) | Tolerability (Retention) |
| --- | --- | --- | --- | --- | --- |
| 1 | Bezafibrate (0.89) | Seladelpar 10mg (0.79) | Seladelpar 200mg (0.86) | Saroglitazar 2mg (0.72) | OCA 10mg (0.86) |
| 2 | Elafibranor 120mg (0.84) | Bezafibrate (0.79) | Elafibranor 120mg (0.81) | Seladelpar 200mg (0.79) | Bezafibrate (0.64) |
| 3 | Elafibranor 80mg (0.75) | Elafibranor 120mg (0.64) | Seladelpar 50mg (0.79) | Seladelpar 50mg (0.66) | Placebo (0.64) |
| 4 | Saroglitazar 2mg (0.70) | Elafibranor 80mg (0.55) | Linerixibat 180mg (0.71) | Seladelpar 10mg (0.58) | OCA 5-10mg (0.54) |
| 5 | Saroglitazar 4mg (0.64) | Seladelpar 50mg (0.50) | Linerixibat 40mg (0.52) | Placebo (0.54) | Elafibranor 120mg (0.48) |
| 6 | Linerixibat (0.51) | Placebo (0.09) | Seladelpar 10mg (0.52) | Linerixibat 40mg (0.54) | Seladelpar 10mg (0.47) |

Supplementary Table 2. Hierarchical Ranking of Therapies Based on P-scores across Multi-dimensional Outcomes

Supplementary Table 2 Legend: Treatment ranking based on P-scores derived from the frequentist network meta-analysis. A higher P-score (ranging from 0 to 1) indicates a higher probability of being the superior treatment for that specific outcome. Note: Seladelpar 10mg exhibits the most balanced profile across efficacy, symptoms, and safety.

| # | Search Query |
| --- | --- |
| 1 | Primary Biliary Cholangitis[Mesh] OR "Primary Biliary Cirrhosis"[Title/Abstract] |
| 2 | PPAR agonists[Mesh] OR "Seladelpar" OR "Elafibranor" OR "Bezafibrate" OR "Fibrates" |
| 3 | FXR agonists OR "Obeticholic Acid" OR "OCA" |
| 4 | IBAT inhibitors OR "Linerixibat" OR "Maralixibat" |
| 5 | Randomized Controlled Trial[Publication Type] |
| 6 | #1 AND (#2 OR #3 OR #4) AND #5 |

Supplementary Table 3. PubMed Search Strategy (Example)

Supplementary Figure S1. Forest Plot for Treatment Discontinuation (Tolerability)

The forest plot shows the Odds Ratios (95% CI) for treatment discontinuation due to adverse events (AEs). Treatments with lower ORs and higher P-scores (e.g., OCA in titrated doses) demonstrate better long-term patient retention compared to those with higher gastrointestinal or pruritic side effects.

Supplementary Figure S2. Multi-dimensional P-score Summary Heatmap

A comprehensive heatmap summarizing the performance of each second-line agent across five key clinical dimensions: Biochemical Response, ALP Normalization, Pruritus Relief, Safety (SAEs), and Tolerability (Retention). The color gradient from red (poor) to green (superior) represents the P-score value (0 to 1).

Supplementary Figure S3. Consistency Assessment via Node-splitting Method

Forest plots comparing direct and indirect evidence for each pairwise comparison in the network. The p-values (all > 0.05) indicate no significant inconsistency, validating the transitivity assumption of the network meta-analysis.

Supplementary Figure S4. Subgroup Analysis by Baseline Disease Severity

Comparison of drug efficacy (Biochemical Response P-scores) between patients with high baseline ALP (> 3x ULN) and those with moderate baseline ALP (< 3x ULN). The analysis explores whether disease severity influences the relative ranking of PPAR and FXR agonists.

Supplementary Figure S5. Individual Risk of Bias Assessment (Traffic Light Plot)

A study-level assessment of the 12 included RCTs using the Cochrane Risk of Bias 2.0 (RoB 2.0) tool. Each circle represents a domain: D1 (Randomization), D2 (Deviations), D3 (Missing data), D4 (Measurement), and D5 (Reporting). Green, yellow, and red colors indicate low risk, some concerns, and high risk, respectively.

Supplementary Figure S6. Overall Risk of Bias Summary (Weighted Bar Chart)

A weighted summary plot showing the percentage of the total evidence base (weighted by sample size) categorized as having a low risk of bias, some concerns, or a high risk of bias across each of the five RoB 2.0 domains.

Supplementary Figure S7. Comparison-adjusted Funnel Plot for Publication Bias

The funnel plot assesses the potential for small-study effects and publication bias for the primary outcome (Biochemical Response). The visual symmetry of the plot, supported by Egger’s test (p > 0.05), suggests no significant evidence of publication bias in the current treatment landscape.
